# Shortwave infrared otoscopy for diagnosis of middle ear effusions: a machine-learning-based approach

**DOI:** 10.1038/s41598-021-91736-9

**Published:** 2021-06-15

**Authors:** Rustin G. Kashani, Marcel C. Młyńczak, David Zarabanda, Paola Solis-Pazmino, David M. Huland, Iram N. Ahmad, Surya P. Singh, Tulio A. Valdez

**Affiliations:** 1 Department of Otolaryngology-Head and Neck Surgery, Stanford University School of Medicine, 801 Welch Road, Palo Alto, CA 94304 USA; 2 Institute of Metrology and Biomedical Engineering, Faculty of Mechatronics, Warsaw University of Technology, Warsaw, Poland; 3 Department of Radiology, Stanford University School of Medicine, Palo Alto, CA USA; 4 Lucile Packard Children’s Hospital, Palo Alto, CA USA; 5 Department of Biosciences and Bioengineering, Indian Institute of Technology Dharwad, Dharwad, Karnataka India

**Keywords:** Translational research, Biomedical engineering, Physical examination, Machine learning

## Abstract

Otitis media, a common disease marked by the presence of fluid within the middle ear space, imparts a significant global health and economic burden. Identifying an effusion through the tympanic membrane is critical to diagnostic success but remains challenging due to the inherent limitations of visible light otoscopy and user interpretation. Here we describe a powerful diagnostic approach to otitis media utilizing advancements in otoscopy and machine learning. We developed an otoscope that visualizes middle ear structures and fluid in the shortwave infrared region, holding several advantages over traditional approaches. Images were captured in vivo and then processed by a novel machine learning based algorithm. The model predicts the presence of effusions with greater accuracy than current techniques, offering specificity and sensitivity over 90%. This platform has the potential to reduce costs and resources associated with otitis media, especially as improvements are made in shortwave imaging and machine learning.

## Introduction

Otitis media refers to inflammation of the middle ear and encompasses a spectrum of pathology ranging from acute otitis media (AOM) and otitis media with effusion (OME) to chronic suppurative otitis media^1^. AOM and OME are widely prevalent, with an annual global incidence of 10.9%, amounting to 709 million cases with 51% occurring in children under age five^2^. The presence of an effusion in the middle ear, which is a sterile fluid collection in the setting of OME, as opposed to an acute infection in AOM, is characteristic to both of these conditions^3^.

Children are particularly susceptible to developing middle ear effusions due to the morphology of their eustachian tube and frequent exposure to viral respiratory pathogens^4^. Viral inflammation can impair mucociliary clearance and predispose to translocation of nasopharyngeal bacteria, such that AOM and OME are seen in 37% and 24% of patients after viral illness^5^. Worldwide, *Streptococcus pneumoniae*, non-typeable *Haemophilus influenzae*, and *Moraxella catarrhalis* are the most dominant organisms^6,7^.

Otitis media, though exceedingly common, is not a benign disease process. AOM carries risk of intracranial complications such as meningitis and brain abscess^8^, as well as extracranial complications like acute mastoiditis, subperiosteal abscess formation, and facial nerve paralysis^9^. In the pre-antibiotic era, intracranial complications were seen in up to 6.4% of cases with 76.4% mortality^10^. Though intracranial complications have since become quite rare but are still fatal in up to 18.6% of cases^11^. Persistent effusions seen in OME cause hearing loss, impairing speech and language acquisition^2^. There is no effective medical treatment for OME, often requiring a surgical procedure, placement of tympanostomy tubes, which is currently the most commonly performed ambulatory surgery in the United States^12^.

The most reliable method for diagnosis of otitis media in clinical practice is pneumatic otoscopy, which provides visible light illumination and mobility assessment of the tympanic membrane (TM)^13^. However, the accuracy of pneumatic otoscopy is subject to user-dependent variability, as a hermetic seal must be established in the ear canal and otoscopic findings can be subtle^14–17^. This variability results in discrepancies in the ability for pediatricians and otolaryngologists to correctly identify middle ear effusions at a rate of 51% and 73%, respectively^18,19^. While tympanometry is a useful adjunct to otoscopy, it is typically unavailable in the primary care setting with varying accessibility in otolaryngology clinics^13^.

Given the possible complications of untreated disease, many practitioners will frequently prescribe antibiotics despite equivocal findings on otoscopy^20^. The widespread use of antibiotics for otitis media has engendered drug resistant strains of the common causative pathogens^21^. As a result, oral cephalosporins, trimethoprim-sulfamethoxazole, and macrolides have lost their efficacy over time^22^. In addition to antibiotic stewardship, correct initial diagnosis of AOM can reduce the incidence of adverse events from antimicrobials, rash and diarrhea being most frequent^23^. It is estimated that antibiotics are given without indication in 32% of cases^24^.

Improving the ability to detect middle ear effusions is thus of considerable interest. Different imaging techniques are under investigation including digital otoscopy, anti-confocal spectroscopy, optical coherence tomography, and ultrasound^25–31^. Prior work by our group suggests that shortwave infrared (SWIR) otoscopy, which uses light with a wavelength of 1000–2000 nm, may enhance detection of middle ear effusions^32^. In contrast to visible light, SWIR light penetrates the tympanic membrane more readily and is also heavily absorbed by water^33^. As a result, SWIR otoscopy provides sharper contrast between fluid-filled and aerated portions of the middle ear, as well as greater visualization of middle ear structures^34^. SWIR images are also amenable to computational image processing and learning techniques, meaning there is potential to automate the diagnostic process via machine learning^35–37^.

Automated detection of middle ear effusions with machine learning would obviate the need for a practitioner to interpret images and has potential to reduce inappropriate antibiotic use, improve patient care outcomes, and cut healthcare costs associated with otitis media. Machine learning is an application of artificial intelligence designed to predict or diagnose novel situations from prior observations. Machine learning models use an algorithm that is repeatedly applied to analyze a data set, recognizing patterns in the data during the training process. Eventually a deep learning model can be designed where the algorithm can predict the presence of a disease state without human input. Deep learning techniques are also widely used for image processing and classification^38–42^. In recent years, investigators have studied machine learning to automate diagnosis in the field of otolaryngology, especially with respect to otitis media^43–45^.

The present study combines a novel imaging technology for otitis media with machine learning approaches in order to (1) distinguish between ears with and without effusions using SWIR otoscopy images, and (2) automate the analysis of images using minimal manipulation of raw data. The technical details of a current prototype SWIR otoscope and study protocol are presented.

## Methods

### Otoscope design

**Figure 1:**
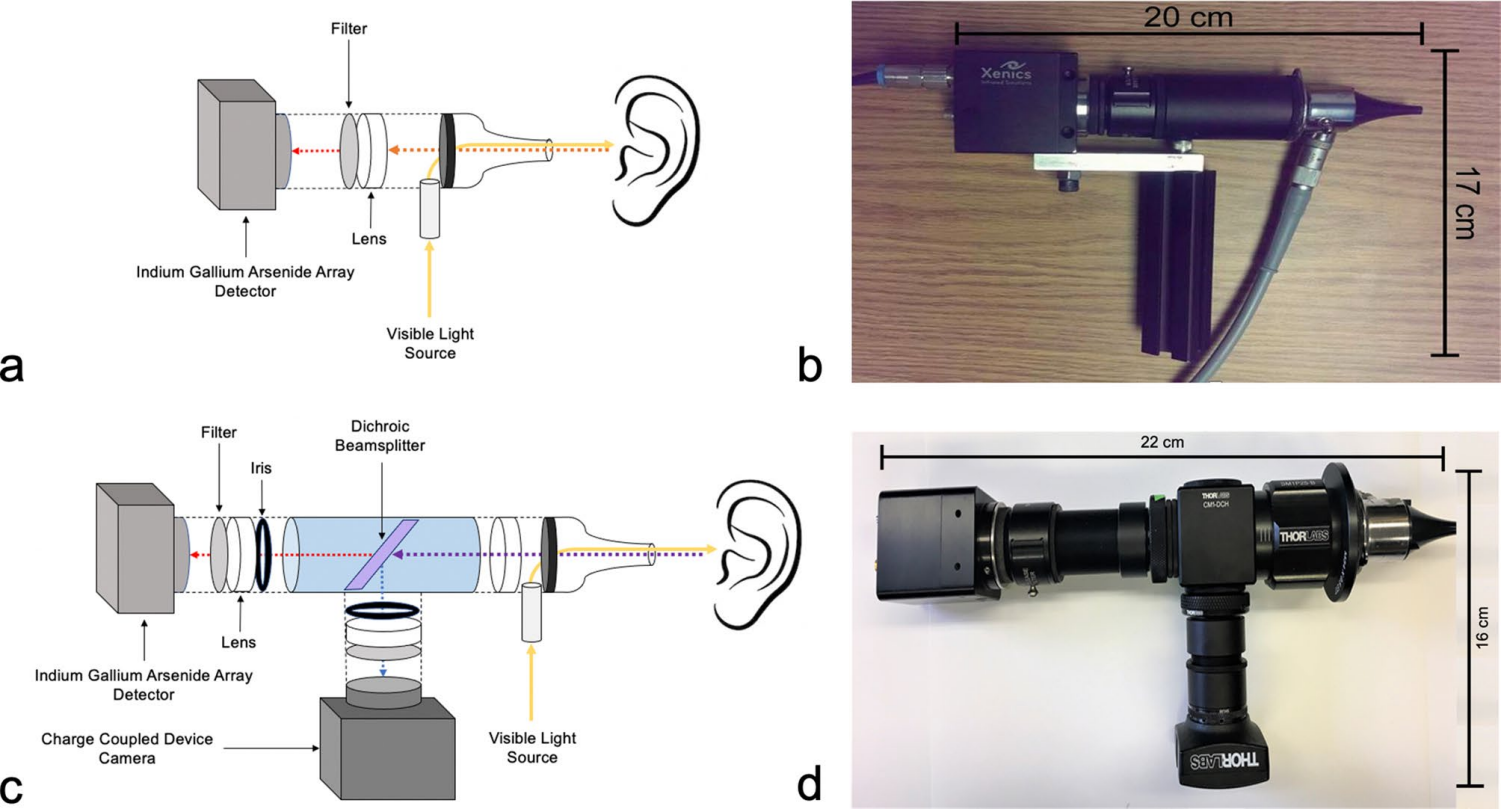
Prior and current iterations of the prototype shortwave infrared (SWIR) otoscope. (**a**) Schematic of the prior SWIR otoscope used by our group. Light is reflected off the tympanic membrane into a lens, then through a 1300 nm long pass filter into a SWIR light detector array, (**b**) image of prior prototype otoscope, (**c**) schematic of current prototype SWIR otoscope, featuring simultaneous visible light and SWIR capturing due to incorporation of a dichroic beam-splitter, finer tuning of exposure with adjustable iris diaphragms, and improved ergonomics, (**d**) image of current prototype otoscope.

The current and prior iterations of the SWIR otoscope are presented in Fig. 1. Compared to our previous SWIR otoscope, the new prototype allows the user to simultaneously capture visible and SWIR images, features a more ergonomic design, and incorporates iris diaphragms for fine tuning of image quality. Briefly, this system consists of a broadband halogen light source (Thorlabs, New Jersey USA, #OSL2) coupled to a medical speculum (Welch Allyn, New York USA, #52133) used to guide the device into the external auditory canal. Diffusely reflected light then passes through a 825 nm dichroic beam splitter at 45 degrees (Semrock, New York USA, #FF825-SDi01-25 × 36 × 2.0). Light with wavelength above 825 nm is reflected off the beam splitter, passing through an adjustable iris diaphragm and a 1300 nm long-pass filter. The SWIR light is then focused onto an Indium Gallium Arsenide (InGas) array detector (Raptor Photonics, Larne UK, #OW1.7-VS-CL-640-II) using 25.4 mm diameter lenses with focal length of 250 mm (Thorlabs, #LB1056). The detector has a 320 × 256 array of 30 µm pixels and 14 bit analog-to-digital conversion, operating with sensor temperature of 280 K. Light with wavelengths of 825 nm or shorter pass through the beam splitter, again through an iris diaphragm and focusing lenses, into a C-mounted charge-coupled camera (Thorlabs, #DCU223C) with 1024 × 768 resolution. The dichroic lens is mounted using a 30 mm cage cube. The entire device along with the optics and camera measures 22 cm in length with a 16 cm handle. The entire apparatus weighs 500 g. It must be connected to a computer for data collection as well as the separate halogen light source.

### Otoscopy protocol

Thirty pediatric patients undergoing tympanostomy tube placement at Lucile Packard Children’s Hospital from 2018 to 2020 were recruited as part of a study (#44,549) approved by the Institutional Review Board of Stanford University. All patients provided informed written consent and study protocols were in accordance with the Declaration of Helsinki guidelines. Otoscopy was performed intraoperatively after induction of anesthesia. The SWIR otoscope was introduced into both the right and left external auditory canal (EAC) to visualize the tympanic membrane in each patient. Different size specula could be used to accommodate anatomic variation in the size of the EAC. A loop curette was used to remove any obstructive cerumen if present. The visible light camera was used to advance the otoscope within 2 cm of the tympanic membrane. Upon optimal placement, visualization was switched over to the SWIR camera and recordings were taken for sixty seconds. The otoscope was then withdrawn. Tympanostomy tube placement followed, and the presence or absence of middle ear fluid was confirmed upon myringotomy. Myringotomy is the gold standard to determine if an effusion is present, as incising the tympanic membrane provides direct visualization of any fluid emanating out from the middle ear space.

### Image analysis

Image analysis was performed in several steps. Preprocessing was performed first, in which images were manually annotated to exclude artifact and identify the region of interest (ROI) in each frame. Automatic segmentation and parameterization followed to produce a binary mask of each frame and identify input features. A de novo deep learning model was devised to make predictions based on these input features, using simple thresholding and Random Forest techniques for classification.

**Figure 2:**
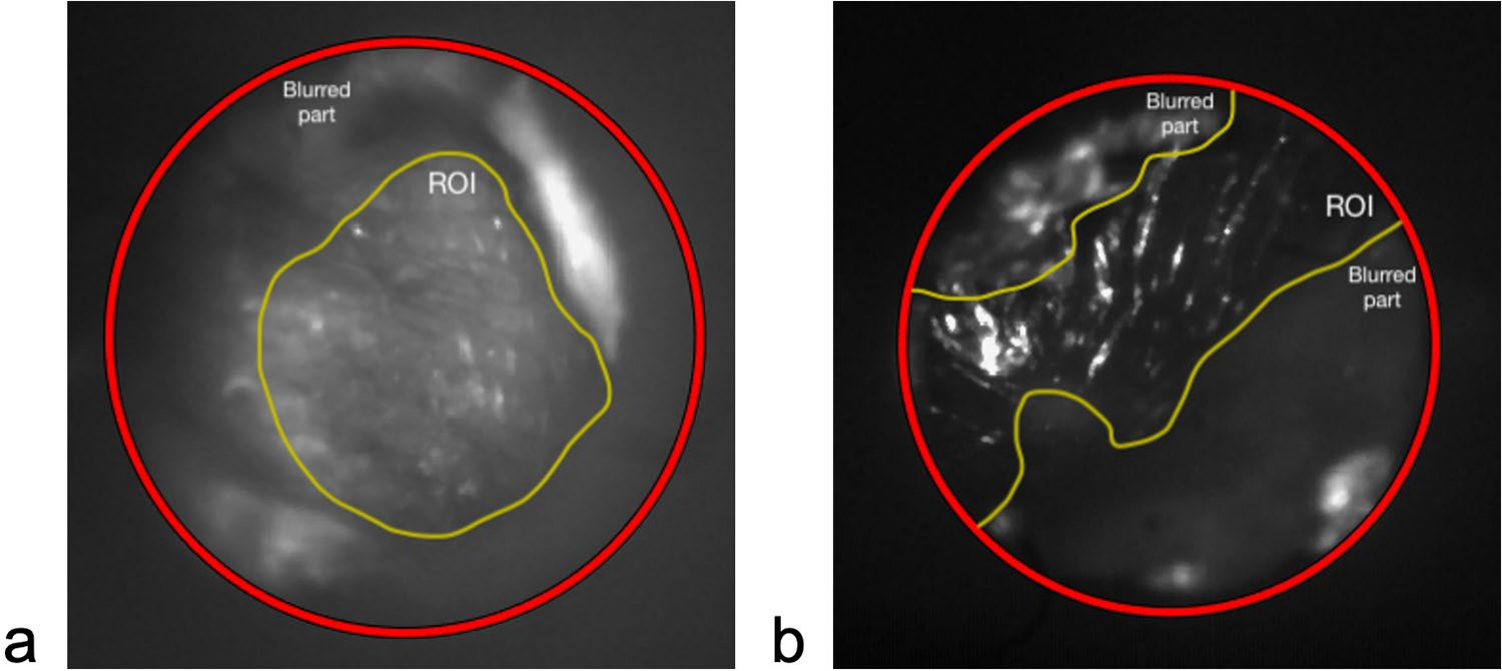
Examples of manual image review, where each frame of the shortwave infrared otoscopy video was annotated to label artifacts such as cerumen, hair, and non-focused regions. (**a**) Portion of tympanic membrane is non-focused, bounded between the red and yellow lines, (**b**) another example of annotations performed to exclude non-focused regions of the tympanic membrane.

Preprocessing and segmentation involved manual review of the SWIR otoscopy images. Each 60 s video was reviewed in a blinded fashion by an otolaryngologist frame by frame. First, the most representative single frame of the tympanic membrane was identified. This was termed “best frame”. Second, an image sequence consisting of 20–22 consecutive frames was randomly selected within each video, termed “frame range”. Third, for each “best frame” and “frame range”, the region of interest (ROI) within each frame was identified. This was performed by excluding regions of artifact such as cerumen, hair, or out of focus regions, leaving only the focused representative portions of the tympanic membrane. A total of 1179 frames were manually annotated. Figure 2 provides an illustration of the ROI and annotations performed.

Several approaches to automatic segmentation were tested. These included circle selections using Hough transform static thresholding, adaptive thresholding, K-means segmentation, active contours, and blurred parts detection. However, these methodologies were only able to find pixels outside the ROI, meaning they were highly specific but lacked adequate sensitivity.

**Figure 3:**
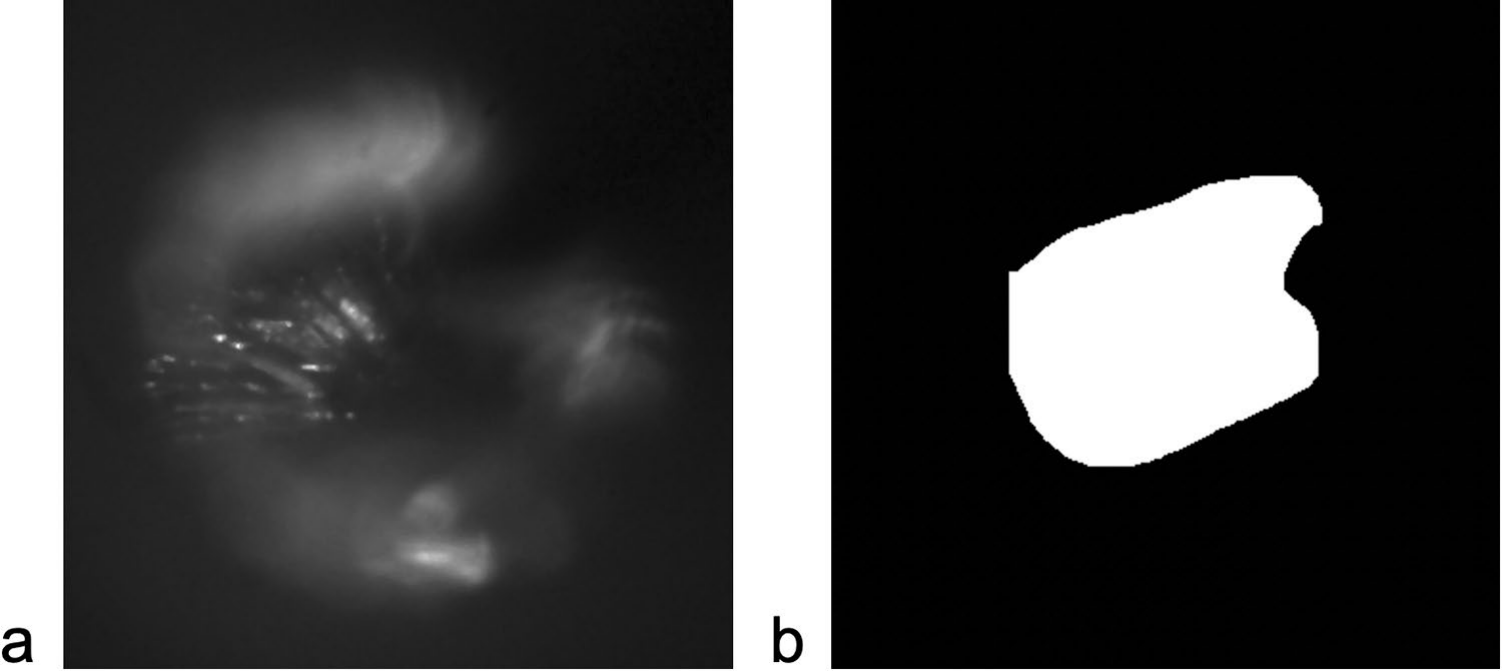
Sample image undergoing semantic segmentation. (**a**) Example of an input taken from a single SWIR frame, (**b**) output image as a binary mask after segmentation. The white portion indicates the region of interest.

Therefore, we tested deep-learning-based semantic segmentation to enhance the predictive power of the program. This method can take red–green–blue (RGB) or grey images as input and produce segmented fields of individual classes as output. An example of an input for a single frame, and the output (binary mask after segmentation) is presented in Fig. 3. There were only two output classes—(1) belonging to the ROI and (2) not belonging to the ROI.

All operations were performed using MATLAB R2019A (Mathworks, Natick MA, https://www.mathworks.com/products/matlab.html). The network was built using the *segnetLayers()* MATLAB function. without using pre-trained weights values, with an encoder depth of 3. Input image size was matched to 128 × 160. The training option selected was the stochastic gradient descent with momentum of 0.9. Data augmentation was implemented with ± 15 pixels in each direction. There were 60 epochs, 10,560 iterations, and 20% of all frames were chosen to be a validation subset.

**Figure 4:**
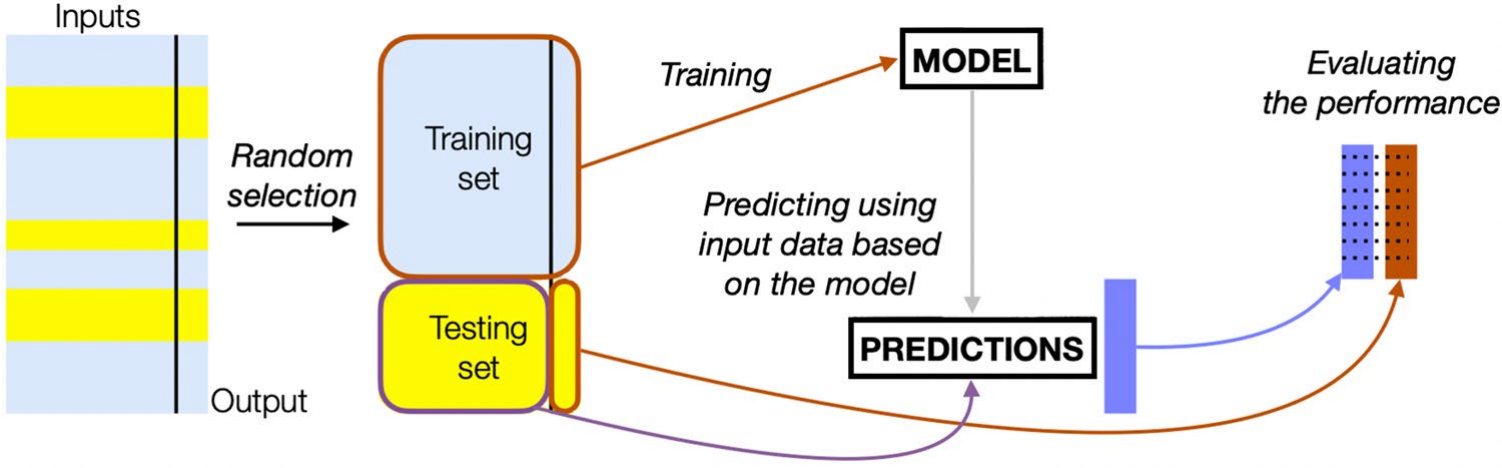
The schematic of the machine learning process after calculating input features inside areas of interest, where input features are automatically selected using the semantic segmentation model.

For all considered frames, the average intensity measure (Input Set 1) inside the region of interest was calculated. Also, other statistical intensity features were calculated, creating Input Set 2. All were treated as global measurements based on the entire region of interest rather than subsections. Apart from average intensity, we included median intensity, mean and median intensities of points being inside the interquartile range (IQR), mean and median intensities of points being greater (or less) than median intensity, and difference in intensities (maximum to median, median to minimum, and IQR). Next, we performed a standard machine-learning scheme (Fig. 4) to create and validate models. We established tenfold hold-out cross-validation with 62/38% division for training and testing subsets, respectively, and ensured the same distribution of two output classes in both subsets.

**Figure 5:**
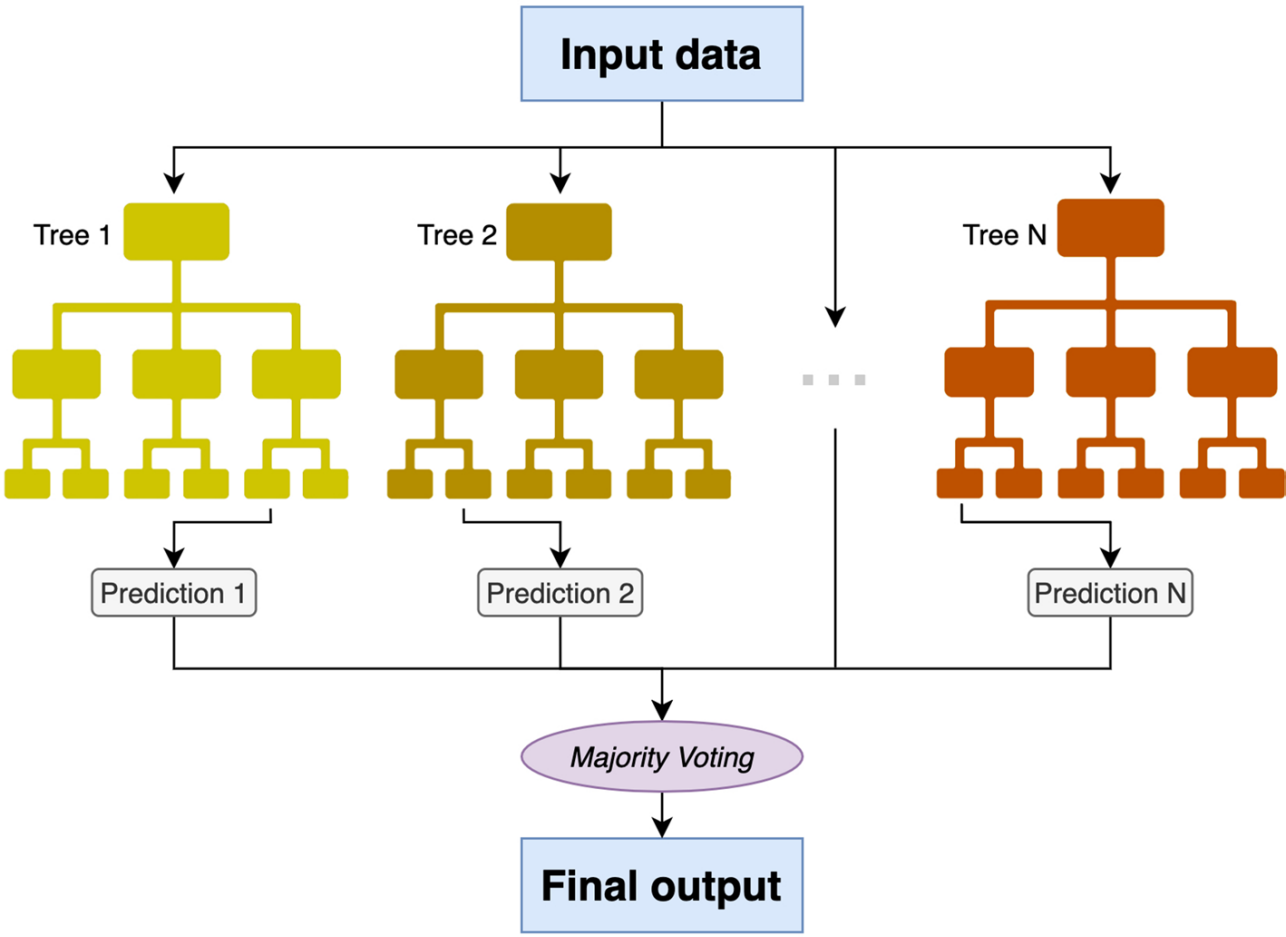
A schematic demonstrating how the final prediction of a random forest classifier is based on input data; N can be any natural number, usually ranging from 50 to 600.

As a model, we evaluated two approaches: simple thresholding using a decision tree and Random Forest methods. Random Forest is an ensemble method (bagging) that operates by constructing a great number of decision trees on randomly selected subsets of input data and establishing the output by averaging all predictions from the individual trees. The schematic presentation of the Random Forest technique is presented in Fig. 5.

The analysis was carried out for 3 scenarios:

- Case 1—Simple thresholding using a decision tree on Input Set 1
- Case 2—Simple thresholding using a decision tree on Input Set 2, to assess the impact of taking more sophisticated input parameters
- Case 3—Random forest with 200 trees on Input Set 2, to assess the impact of using a more advanced classification technique

**Figure 6:**
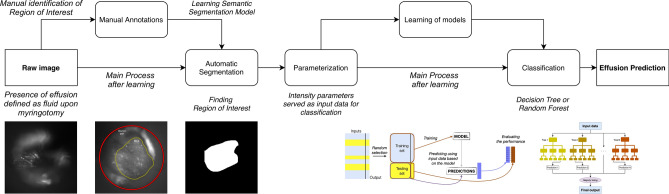
Schematic overview of study protocol. Raw shortwave infrared otoscopic images of the tympanic membrane prior to myringotomy were obtained. An effusion was defined as fluid upon myringotomy. The image frames were manually annotated to identify a region of interest, a process which was then automated. Parameterization followed, using intensity measurements as input data when analyzing each frame. Decision tree and random forest models were then based on the intensity parameters to classify each image and predict the presence of an effusion.

Figure 6 provides an overview of the study protocol starting from the acquisition of raw images to the eventual machine learning prediction.

## Results

A total of 30 patients were recruited for the study. 23 of these patients had OME; 7 had RAOM. A total of 55 ears were examined, as 25 patients had bilateral tympanostomy tube placement and 5 patients had unilateral tube placement. All 55 ears were recorded using the SWIR otoscope. Of these, 19 ears (35%) were found to have a middle ear effusion; 36 (65%) did not. Each 55 SWIR otoscopy recordings were manually reviewed by a blinded otolaryngologist to identify the best frame and frame range in each exam. A total of 1179 frames were manually annotated for each recording to exclude artifact and define the region of interest per frame. Of the 1179 frames, 409 frames (35%) belonged to the effusion group and 770 (65%) frames were examined in the non-effusion group.

**Figure 7:**
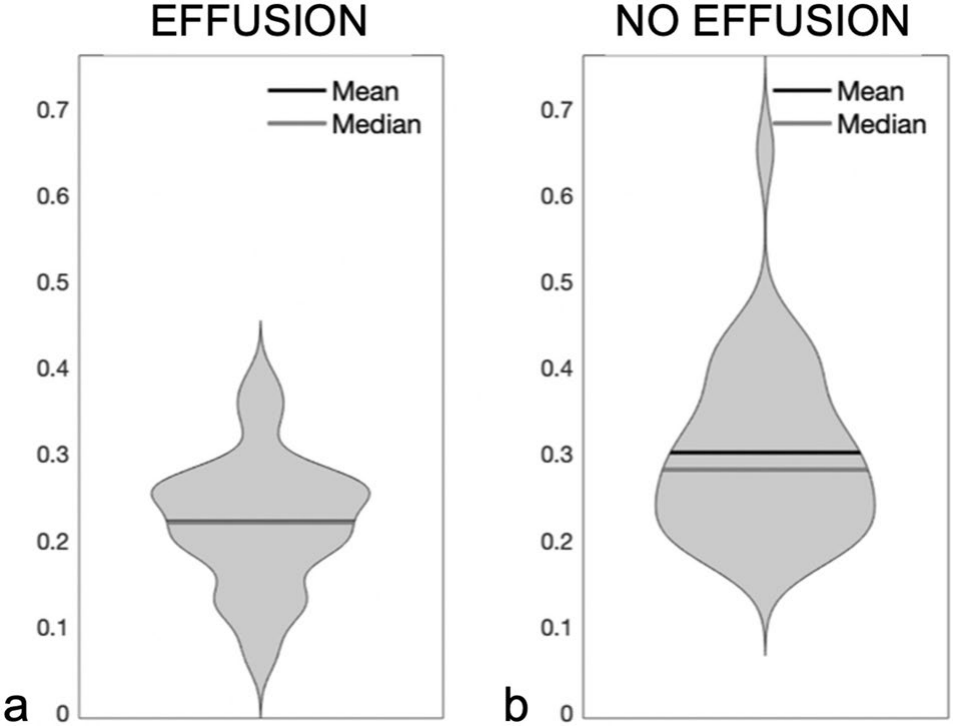
Violin plots presenting the differences in average intensity between ears with middle ear effusions and those without. (**a**) Ears with effusions had average lower intensity compared to (**b**) ears without effusions.

SWIR light is heavily absorbed by fluid, resulting in a darker image of the tympanic membrane when fluid is present in the middle ear. Ears with effusions had lower average intensity compared to effusion-free ears. Thus, average intensity analysis was applied in the form of three machine learning algorithms to process the SWIR images. The first algorithm, Case 1, used average intensity with a decision tree for analysis. The distributions of average intensity values used for Case 1 are presented as violin plots in Fig. 7.

The overlap in the plots suggests that consideration of other parameters could improve effusion discrimination. Case 2 used average intensity and other input measures with a decision tree, and Case 3 incorporated the same parameters as Case 2 but used Random Forest technique rather than a decision tree. The validation accuracy of the semantic segmentation algorithm was 92.4%. The accuracies, sensitivities and specificities of all cases are listed in Table 1.

**Table 1:** The statistical summary of the SWIR otoscopy performance for all considered cases.

	Accuracy	Sensitivity	Specificity
**Case 1** *(Average Intensity* + *Decision Tree)*	69.2%	55.9%	75.8%
**Case 2** *(Set of parameters* + *Decision Tree)*	85.5%	79.4%	88.7%
**Case 3** *(Set of parameters* + *Random Forest)*	90.3%	90.5%	90.1%

**Figure 8:**
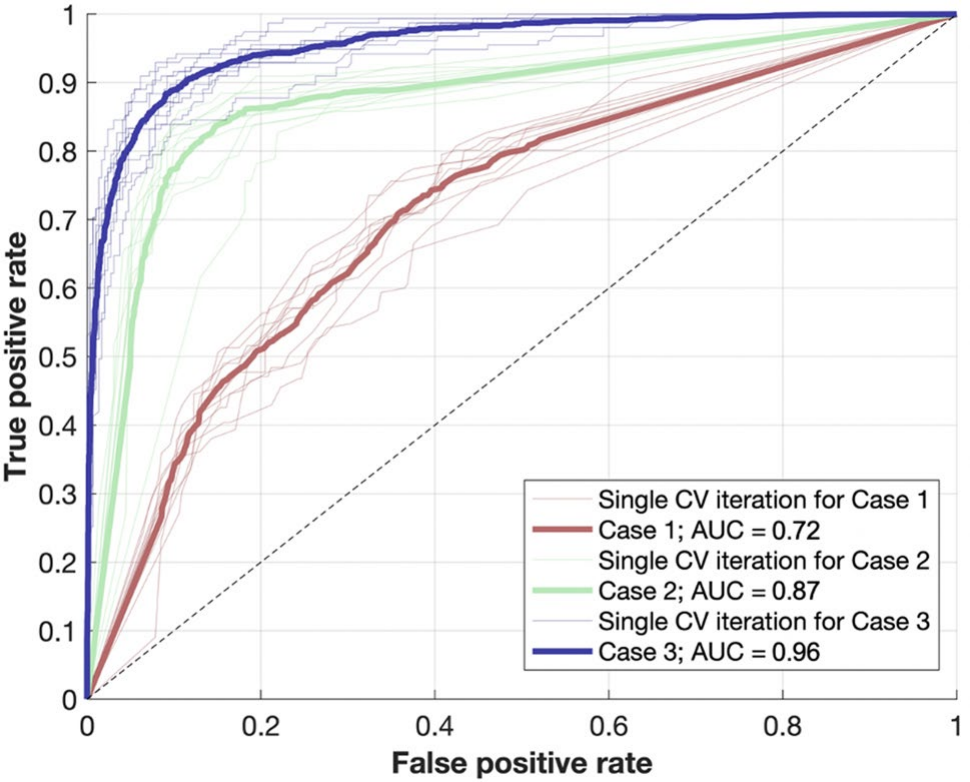
The receiver operator curves representing the performance of the automatic platform for all considered cases; the narrower, transparent lines present results from a single specific iteration of the hold-out cross-validation. AUC = area under curve, CV = cross-validation.

Receiver operator curves were also calculated for each case, as demonstrated in Fig. 8. The individual iterations for each case provided relatively similar results around the average, suggesting good coherence. The shift from Case 1 to Case 2 illustrates the improved diagnostic accuracy from using additional parameters beyond average intensity. Inclusion of an ensemble technique in Case 3 further enhanced sensitivity and specificity, with an area under the curve of 0.96.

## Discussion

Accurate identification of middle ear effusions remains a diagnostic challenge for primary care providers and otolaryngologists alike. As previously mentioned, missing the diagnosis of AOM or OME exacerbates the already significant healthcare burden these conditions carry, with potential for life-threatening complications. This study is the first to apply machine learning algorithms to SWIR otoscopy for automated effusion detection, offering accuracy of 90.3%, sensitivity of 90.5% and specificity of 90.1%. This work builds upon advancements made in both otoscopic imaging and machine learning.

Though first described by Guy De Chauliac in 1363, the modern form of the otoscope traces back to Hartmann in 1881, and only recently have there been efforts to modify otoscopic imaging^46^. Digital otoscopy has gained in popularity over the previous decades but does not appear to hold any diagnostic advantage over traditional otoscopy in identifying effusions^27,47^. This may be due to the inherent limitations of visible light illumination and human interpretation of the images.

A variety of imaging techniques extending beyond the visible light spectrum have shown promise in identifying middle ear pathology. Otoscopy using multi-color reflectance can offer improved assessment of middle ear structures and the tympanic membrane^34,48^. Fluorescent otoscopy can highlight the presence of cholesteatoma with the potential to guide resection attempts in the future^49^. Additionally, an anti-confocal microscopy system has been proposed that may highlight inflammation of the middle ear, though this has yet to be tested in vivo^50^.

Modalities that have been applied to the detection of effusions include low coherence interferometry (LCI), optical coherence tomography (OCT), and SWIR. LCI and OCT are similar techniques that produce cross-sectional imaging using near-infrared light^51^. Investigators have designed an otoscope incorporating LCI and pneumatic otoscopy to predict effusions based on quantification of tympanic membrane mobility^52,53^. Patients with effusions tended to have lower compliance and lower amplitude of TM mobility, though presence of an effusion was not confirmed via myringotomy or tympanocentesis. Multiple studies have been conducted on OCT, showing utility in detecting cholesteatoma, tympanosclerosis, biofilms, and effusions^30,31,54–60^. Recently Preciado and colleagues describe a prototype OCT otoscope which could detect fluid with a sensitivity and specificity of 90.9% and 90.2%, respectively, and differentiate mucoid from serous effusions^61^.

A difficulty shared by all otoscopy modalities is user variability in operation and interpretation. Hence, the ability to automate diagnosis through machine learning has garnered considerable interest. Multiple studies have applied machine learning to visible light otoscopy for identification chronic otitis media with perforation, cerumen, myringosclerosis, and retraction pockets^45,62–64^. Livingstone and colleagues uploaded visible light otoscopy images into a Google Cloud automated algorithm, AutoML, and compared the diagnostic accuracy of the algorithm to physicians from a variety of specialties^44^. Serous otitis media was correctly diagnosed by the algorithm 80% of the time, compared to a rate of 44% among physicians. Another machine learning system using OCT found agreement with physician diagnosis in 91.5% of cases^25^. Manual annotations can also be automated with a machine learning frame selection tool^65^.

Several features of the current SWIR model provide increased diagnostic power. First, operation does not require a pneumatic head, minimizing variability in accuracy due to an insufficient hermetic seal. Second, SWIR light has the ability to contrast fluid and non-fluid filled spaces more effectively than visible light. Third, the deep learning model predicts the presence of fluid without human input, and will become stronger with use of more sophisticated input measures. In the current platform, visual light images were used only to guide the otoscope. We did not apply the presented algorithm to the visual light images because the semantic segmentation model was trained on grey SWIR images, and current parameterization is reasonable only for grey images. However, the algorithm can be expanded to analyze both SWIR and visual light images in the future. To do so, another semantic segmentation model should be trained on RGB images with new manual annotations and new classifiers. Parameters describing RGB color space should also be trained and validated.

We decided primarily to present the process of going from a human-based to machine learning-based approach. This is why we started from a single naïve intensity parameter. Case 1 was treated as a baseline, comprising only a single parameter and a basic classifier – the decision tree, which is representative of how an expert system can operate. We showed that the performance can be improved by extending the set of features that describe the area of interest in the analyzed frame (Case 2). Furthermore, changing the classification model itself (a switch from a single decision tree to the natural extension—so-called “forest” of trees, each trained with randomly selected subsets of training data), improved the accuracy metrics even further (Case 3). We also tested gradient boosted models, but they achieved slightly worse accuracy compared to random forests methods. Neural networks were omitted due to the sample size but will be considered in future studies.

Though an accurate prediction model, several limitations exist. The overall incidence of effusion in the cohort is 35%, resulting in an imbalanced distribution between the effusion and non-effusion groups. One might expect that the majority of ears would have fluid present, given that OME is diagnosed clinically by the suggestion of an effusion. However, the natural history of OME is characterized by a fluctuating course, and spontaneous remission of fluid can be seen at time of myringotomy. Studies using intraoperative myringotomy to assess for fluid have reported effusion incidence of 50–85%, whereas our study found fluid in 35% of ears^66–69^. However, our cohort included 7 children with bilateral RAOM who had no effusion on myringotomy, which would be expected as children with RAOM are disease free between episodes of acute infection – which further lowers the incidence of effusion seen in the study. We anticipate this imbalance would resolve with ongoing data collection, as it is likely a manifestation of limited sample size.

The imbalanced distribution might also result in bias towards the group with more samples. This can be assessed by comparing accuracy and so-called balanced accuracy, which is an average of both sensitivity and specificity. The effect of this imbalance is evident in the decision tree method used for Case 1 and Case 2. However, it is less apparent for the random forest technique used in Case 3, as there were optimized for balanced accuracy where sensitivity is similar to specificity.

Apart from the imbalance, the sample size itself is relatively limited. Therefore, we decided not to divide the data into three subsets by including a validation subset as commonly practiced in neural networks, but rather split the data into training and testing subsets. We also considered applying oversampling approaches; however, as the current results appeared promising, we have opted to gather more data and perform the analysis once more rather than generating artificial data points through oversampling. Furthermore, we decided not to perform a post-hoc calibration of the machine learning models. However, we plan to evaluate sigmoid and isotonic approaches in the larger dataset.

Also, there remains an element of user variability, in that the depth of otoscope insertion can change the light intensity differential between regions of interest and non-interest, and may fail to capture the entire extent of the tympanic membrane. Artifacts such as wax and hair need to either be removed prior to SWIR otoscopy or manually annotated to have a clear view of the area of interest. Subsequent image analysis may suffer if portions of the image are blurry or dark. The mobility of the current prototype otoscope can be improved upon as well. Although the weight of 500 g is similar to conventional otoscopes (350–450 g), it is not yet a portable system as it relies on data collection through a computer and separate light source. Despite the additional bulk, the otoscope was used by multiple practitioners without issue. Future iterations of the design will be more streamlined, with the eventual goal of creating an ergonomic portable system amenable to clinic use.

Had a larger number of ears been examined, the classification chain could have been implemented from raw image to final classification indication, meaning additional deep neural techniques could have been applied. Separate frames were analyzed rather than entire videos because the region of interest did not remain constant from frame to frame. Some frames had darker or unlit portions. Additionally, the algorithm could not recognize which part of the tympanic membrane was presented on each frame. This may affect parameterization in that intensity could change based on technical conditions. Before reaching completely automated video analysis, the next iteration of the algorithm could use representative frame sequences intentionally picked out by an otolaryngologist, rather than the random sequence selection employed currently. We plan to incorporate this modification into future models, which will require a substantially increased sample size.

Future studies can take multiple directions. Creation of a shared SWIR image database would enable investigators to increase the predictive power of machine learning algorithms. Advancements in optics may improve the consistency with which an entire frame is in focus. The efficacy of the program could be assessed using visible light images as a reference, in addition to SWIR images. Finally, the program should be verified against otoscopic SWIR images that were outside the database used for the machine learning procedure.

## Data Availability

Data and materials used in this study are available upon reasonable request to the corresponding author and under a collaboration agreement.

## Code availability

Computer code used in this study are available upon reasonable request to the corresponding author and under a collaboration agreement.
